# Comparative analyses of three swallow species (Aves, Passeriformes, Hirundinidae): Insights on karyotype evolution and genomic organization

**DOI:** 10.1590/1678-4685-GMB-2019-0232

**Published:** 2020-03-09

**Authors:** Suziane Alves Barcellos, Rafael Kretschmer, Marcelo Santos de Souza, Alice Lemos Costa, Tiago Marafiga Degrandi, Cassiane Furlan Lopes, Malcolm A. Ferguson-Smith, Jorge Pereira, Edivaldo Herculano Correa de Oliveira, Ricardo José Gunski, Analía del Valle Garnero

**Affiliations:** ^1^ Universidade Federal do Pampa, Programa de Pós-graduação em Ciências Biológicas - PPGCB, São Gabriel, RS, Brazil.; ^2^ Universidade Federal do Rio Grande do Sul, Programa de Pós-graduação em Genética e Biologia Molecular - PPGBM, Porto Alegre, RS, Brazil.; ^3^ Universidade Federal do Paraná, Programa de Pós-Graduação em Genética, PPGG, Curitiba, PR, Brazil.; ^4^ University of Cambridge Department of Veterinary Medicine, Cambridge Resource Centre for Comparative Genomics, Cambridge, United Kingdom.; ^5^ Universidade Federal do Pará, Instituto de Ciências Exatas e Naturais, Belém, PA, Brazil.; ^6^ Instituto Evandro Chagas, Laboratório de Cultura de Tecidos e Citogenética, Ananindeua, PA, Brazil.

**Keywords:** Homology, molecular cytogenetics, fluorescent *in situ* hybridization, Hirundinidae

## Abstract

Despite the richness of species in the Hirudinidae family, little is known about the genome organization of swallows. The *Progne tapera* species presents genetic and morphological difference when compared to other members of the same genus. Hence, the aims of this study were to analyze the chromosomal evolution of three species *Progne tapera*, *Progne chalybea* and *Pygochelidon cyanoleuca* - by comparative chromosome painting using two sets of probes, *Gallus gallus* and *Zenaida auriculata*, in order to determine chromosome homologies and the relationship between these species. All karyotypes exhibited 76 chromosomes with similar morphology, except for the 5th, 6th and 7th chromosome pairs in *P. cyanoleuca*. Additionally, comparative chromosome painting demonstrated the same hybridization pattern in the two *Progne*, which was similar to the putative avian ancestral karyotype, except for the centric fission in the first pair, as found in other Passeriformes. Thus, these data display a close relationship between the *Progne* species. Although *P. cyanoleuca* demonstrated the same fission in the first pair of the ancestral syntenic (GGA1), it also showed an additional chromosomal rearrangement for this species, namely a fusion with a microchromosome in the seventh pair.

## Introduction

The order Passeriformes is one the most diverse within the class Aves, including around 6000 species (Del Hoyo *et al.*, 2011). As the other members from this class, it presents small genome, high chromosomal number, a few pairs of macrochromosomes and several microchromosome pairs. Furthermore, birds have a sexual system ZZ/ZW, where the female is heterogametic (Griffin *et al.*, 2007; Barcellos *et al.*, 2019).

The Hirundinidae family (Aves: Passeriformes), commonly known as swallows, comprises approximately 84 species (Dickinson, 2003; Sheldon *et al.*, 2005). These birds are well known worldwide due to their cosmopolitan habits, behavior and ecology (Sheldon *et al.*, 2005). Moreover, they are migratory and insectivorous. Due to the scarcity of food resources in winter, swallows tend to fly several miles to find food and a safe place to stay during this season (Sigrist, 2013).

*Progne tapera* (Linnaeus, 1766)*, Progne chalybea* (Gmelin, 1789) and *Pygochelidon cyanoleuca* (Vieillot, 1817) have similar karyotypical organization with the same diploid number (2n=76) and distribution of repetitive DNA. Furthermore, recent studies with these species found an interesting characteristic, an enlarged W chromosome (Barcellos *et al.*, 2019). Despite recent research, the cytogenetics of swallows is still poorly defined.

Cross-species chromosome painting has been applied widely for evolutionary biology studies and karyotype evolution (Ferguson-Smith and Trifonov, 2007; Ellegren, 2010) and, in particular, to identify chromosomal homologies in passerines species (Kretschmer *et al.*, 2018a). The most common probes used for analyses in birds are from *Gallus gallus* (Linnaeus, 1758) (GGA) and *Leucopternis albicollis* (Latham, 1790) (LAL) (Griffin *et al.*, 2007; de Oliveira *et al.*, 2010). Using GGA and LAL probes in Passeriformes revealed a fission and numerous inversions in the first chromosome pair of the ancestral syntenic (GGA1), an apomorphy that has been seen in all species belonging to this order analyzed by fluorescent *in situ* hybridization (FISH) (Kretschmer *et al.*, 2014, 2015, 2018a; dos Santos *et al.*, 2015, 2017).

Recently, a new set of whole chromosome-specific probes was developed using *Zenaida auriculata* species (Des Murs, 1847). This probe set has the similar organization pattern of macrochromosomes as the putative ancestral karyotype of birds (PAK) and is also similar to that of *G. gallus*. Furthermore, it shows interchromosomal rearrangements, which are extremely important for karyotype evolution (Kretschmer *et al.*, 2018b).

In order to study the genome organization of Hirundinidae, and the relationship between them, we present here for the first time chromosome painting using whole-chromosome probes of *G. gallus* and *Z. auriculata* in three species of swallows, *P. chalybea, P. tapera* and *P. cyanoleuca*.

## Material and Methods

### Species

The present work examines twelve individuals belonging to the Hirundinidae family: *P. chalybea* (3 females and 2 males); *P. tapera* (2 females and 2 males) and *P. cyanoleuca* (3 females), all collected in São Gabriel – Rio Grande do Sul State, Brazil (SISBIO Permission Number: 33860-4). The protocols were approved by the Ethics Committee on the use of animals (CEUA- Universidade Federal do Pampa, 026/2012).

### Chromosome isolation

Chromosomes were obtained by fibroblast culture (Sasaki *et al.*, 1968) and short-term bone marrow culture (Garnero and Gunski, 2000). The procedures included: hypotonic treatment, incubation with colchicine (0,05%) and cell fixation using methanol and acetic acid (3:1). Diploid number and chromosome morphology were determined from the analyses of 30 mitotic cells stained with Giemsa 5% in 0.07 M phosphate buffer, pH 6.8. Subsequently, metaphases were analyzed by microscopy.

### Fluorescent *in situ* hybridization (FISH)

Chromosome painting utilized two sets of probes: *Z. auriculata* (ZAU 1-8 and Z) and *G. gallus* (GGA 9-10). Comparisons were based on homology between ZAU and GGA (Kretschmer *et al.*, 2018b). Protocols for hybridization were performed as described in de Oliveira *et al.* (2010). The FISH results were examined by epifluorescent microscopy.

## Results

### Karyotype analyses

**Figure 1:**
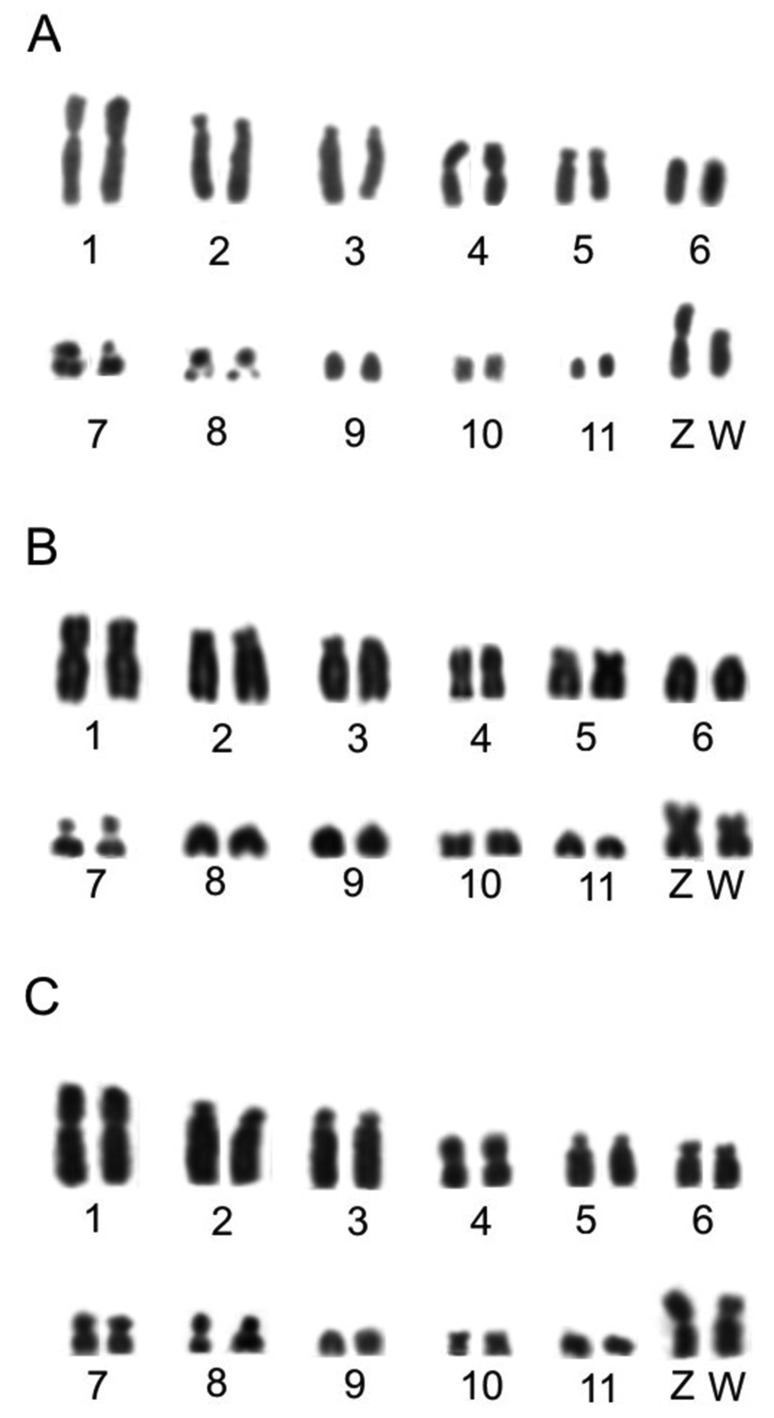
Partial karyotype of three females with conventional staining (Giemsa). A= *P. tapera*, B= *P. chalybea* and C= *P. cyanoleuca.*

The diploid number is 76 for the three swallow species, which corroborate previous studies (Barcellos *et al.*, 2019). The 1st, 4th, 10th pairs and also the Z chromosome are metacentric, pairs 2 and 3 are acrocentric, while 8th, 9th, 11th and all microchromosomes are telocentric. Only three morphological differences between species were observed: in *P. cyanoleuca,* the 5th chromosome pair is acrocentric, the 6th submetacentric, and the 7th metacentric, whereas in the *Progne* species the 5th pair is submetacentric and the 6th and 7th chromosome pairs are telocentric. The W sex chromosome is submetacentric in all the three species (Figure 1).

### Chromosome painting using GGA and ZAU probes

**Figure 2:**
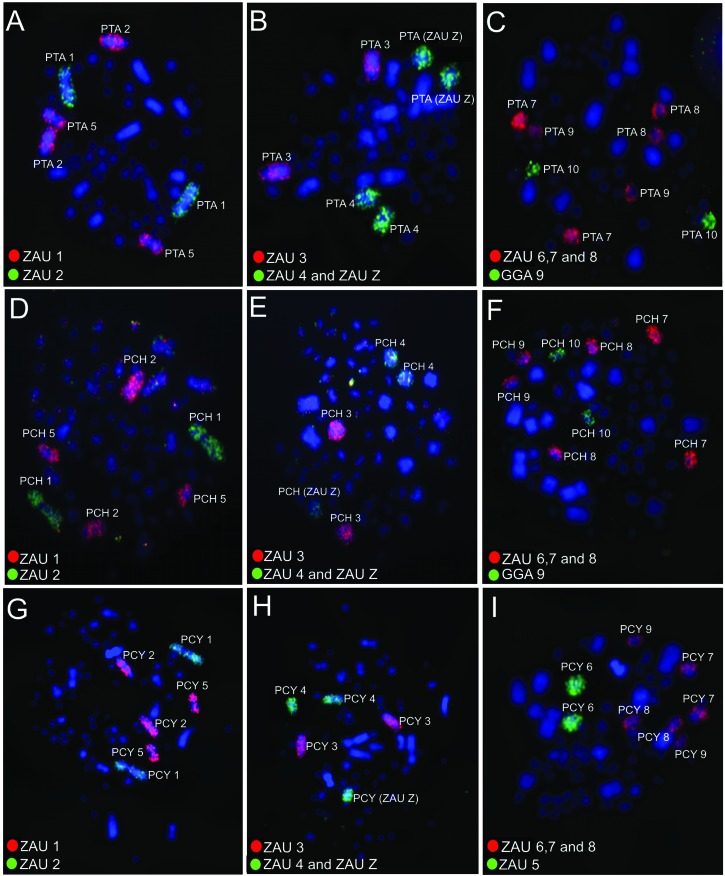
FISH experiments using *Gallus gallus* (GGA) and *Zenaida auriculata* (ZAU) probes hybridized onto *P. tapera* - PTA (A-C), *P. chalybea* - PCH (D-F) and *P. cyanoleuca -* PCY (G-I) metaphases. Probes are indicated in the lower left corner of each image; probes were labeled with biotin/Cy3 (red) or fluorescence (green).

**Figure 3:**
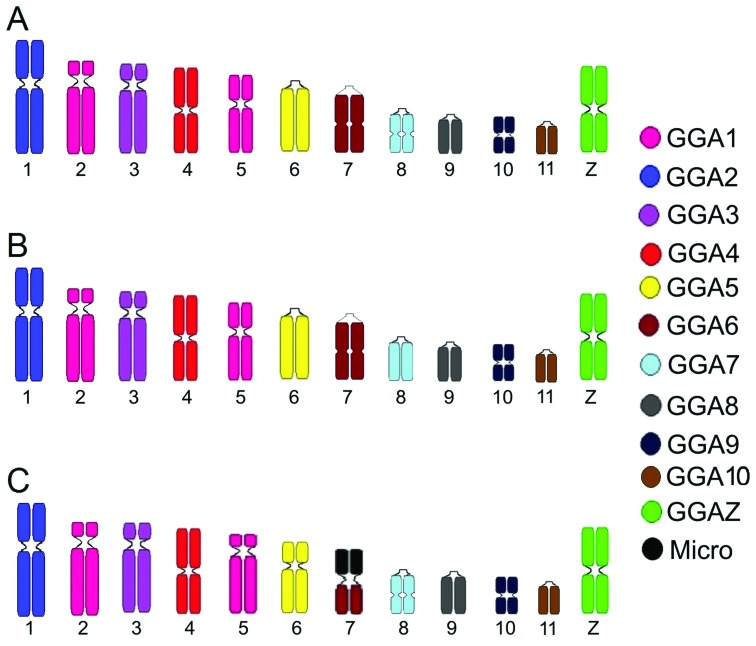
Homology map between *G. gallus* and three swallow species: *P. tapera* (A) and *P.chalybea* (B), *P. cyanoleuca* (C).

GGA and ZAU probes revealed conservation of some syntenic groups in swallows. ZAU1 hybridized in the second and fifth chromosome pairs, while ZAU2 demonstrated hybridization signals only in the first pair in the three swallow species (Figure 2 A, D and G). The hybridization patterns using GGA and ZAU probes were the same for *P. tapera and P. chalybea*. Hybridization experiments demonstrated that GGA1 (ancestral chromosome 1) corresponds to two distinct chromosome pairs and GGA2, 3, 4, 5, 6, 7, 8, 9 and 10 each correspond to one pair in swallows (Figure 3 A and B). *P. cyanoleuca* exhibited a similar pattern of hybridization, except for the seventh pair of chromosomes, which shows fusion with a microchromosome (Figures 2 I and 3 B).

## Discussion

The swallow karyotypes presented a diploid number and morphology typically found in most Passeriformes (Tegelström and Ryttman, 1981; Santos and Gunski, 2006), with 76 chromosomes and many microchromosome pairs, as observed in Barcellos *et al.* (2019). However, these species have a W chromosome relatively bigger than the W of most Passeriformes species due to a highly repetitive sequences accumulation, such as microsatellites (Berlin and Ellegren, 2004; Chen *et al.*, 2012; Zhang *et al.*, 2014; Barcellos *et al.*, 2019).

The Neognathae group, so called modern aves, is divided in two distinct clades: Galloanseres and the Neoaves. *G. gallus* belongs to Galloanseres, considered a basal group and very similar to PAK. On the other hand, *Z. auriculata* is a Neoaves member, so it is closer to derived groups (Jarvis *et al.*, 2014). Despite the phylogenetic distance, there is a high similarity between GGA and ZAU. Since studies using both probes showed the same chromosomal homologies in both, except for GGA4, which has a fusion between two ancestor chromosomal pairs (PAK4+10), while ZAU shows fission (ZAU4-11) (Kretschmer *et al.*, 2018b). However, the use of ZAU probes showed clear hybridization signals in derived species (Kretschmer *et al*., 2020), which can also be observed in the swallows, probably due to their phylogenetic proximity.

The main differences found when compared to the putative ancestral avian karyotype (Griffin *et al.*, 2007), is the fission in the first chromosome pair into two distinct pairs of macrochromosomes (2 and 5 pairs) and the fusion of a microchromosome in the seventh pair in *P. cyanoleuca*. The fission of the GGA 1 chromosome has been observed in all Passeriformes studied by FISH so far (Guttenbach et al., 2003; Derjusheva et al., 2004; Kretschmer et al., 2014, 2015, 2018a; dos Santos et al., 2015). In addition, this rearrangement can also be seen in most Accipitriformes, as well as in Psittaciformes (Kretschmer *et al.*, 2018a), suggested as Passeriformes sister group (Hackett *et al.*, 2008; Jarvis *et al.*, 2014).

Among the swallows analyzed by FISH, only one chromosomal rearrangement was observed, a fusion with a microchromosome pair in *P. cyanoleuca*, which is the most derived species. This fusion had possibly occurred due to evolutionary pressures leading to interchromosomal and intrachromosomal rearrangements (Ellegren, 2010). Fusions among macrochromosomes and microchromosomes were also observed in two species of the genus *Falco* (Accipitriformes) (Kretschmer *et al.*, 2018a). Although highly variable among lineages, intrachromosomal rearrangements might be significant contributors to the genetic and phenotypic diversity presented by the members of the class Aves (Damas *et al.*, 2019).

The chromosome painting patterns found in swallows are very distinct when compared to distant bird orders, such as Galliformes and Anseriformes, which in general present chromosomal structure similar to PAK1-10 (Guttenbach *et al.*, 2003; Shibusawa *et al.*, 2003; Kretschmer *et al.*, 2018a). The same occurs when compared with Gruiformes, Eurypygiformes and Trogoniformes, which show fusions in distinct macrochromosomes pairs and also some peculiar chromosomal fission for each order (Nanda *et al.*, 2011; Furo *et al.*, 2015; Degrandi *et al.*, 2017).[Bibr B29]

In the past, the Hirundinidae family had some phylogenetic issues in relation to *P. tapera* due to the phenotypic and genetic difference from other *Progne* species (Moyle *et al.*, 2008). Nevertheless, recent studies using classical and molecular cytogenetics have shown a greater similarity between the *P. tapera* and *P. chalybea* when compared to *P. cyanoleuca* (Barcellos *et al.*, 2019). Taken together, these examples and our data support the current phylogeny of the genus *Progne*, which puts all *Progne* species into a single genus (Sheldon et al., 2005; Moyle et al., 2008; Barcellos et al., 2019).

Overall, the analyses allow us to identify homologies between PAK and three swallow species using GGA and ZAU probes, providing data about the mechanisms involved in karyotype evolution in the Hirundinidae family. Moreover, FISH experiments played an important role in identifying chromosomal rearrangements, such as the microchromossome and macrochromosome fusion in *P. cyanoleuca* species, which clarifies the relationships among the swallows.

## References

[B1] Barcellos SA, Kretschmer R, de Souza MS, Costa AL, Marafiga TD, dos Santos M, de Oliveira EHC, Cioff MB, Gunski RJ, Garnero ADV (2019). Karyotype evolution and distinct evolutionary history of the W chromosomes in swallows (Aves, Passeriformes). Cytogenet Genome Res.

[B2] Berlin S, Ellegren H (2004). Chicken W: a genetically uniform chromosome in a highly variable genome. Proc Natl Acad Sci U S A.

[B3] Chen N, Bellott DW, Page DC, Clark AG (2012). Identification of avian W-linked contigs by short-read sequencing. BMC Genomics.

[B4] Damas J, O’Connor RE, Griffin DK, Larkin DM, Kraus RHS (2019). Avian chromosomal evolution. Avian Genomics in Ecology and Evolution.

[B5] de Oliveira EHC, Tagliarini MM, Rissino JD, Pieczarka JC, Nagamachi CY, O’Brien PCM, Ferguson-Smith MA (2010). Reciprocal chromosome painting between white hawk (*Leucopternis albicollis*) and chicken reveals extensive fusions and fissions during karyotype evolution of Accipitridae (Aves, Falconiformes). Chromosome Res.

[B6] Degrandi TM, Garnero ADV, O’Brien PCM, Ferguson-Smith MA, Kretschmer R, de Oliveira EHC, Gunski RJ (2015). Chromosome painting in *Trogon s. surrucura* (Aves, Trogoniformes) reveals a karyotype derived by chromosomal fissions, fusions, and inversions. Cytogenet Genome Res.

[B7] Del Hoyo J, Elliott A, Christie AD (2011). Handbook of the Birds of the World.

[B8] Derjusheva S, Kurganova A, Haberman F, Gaginskaia E (2004). High chromosome conservation detected by comparative chromosome painting in chicken, pigeon and passerine birds. Chromosome Res.

[B9] Dickinson EC (2003). The Howard & Moore complete checklist of the birds of the world.

[B10] dos Santos MDS, Kretschmer R, Frankl-Vilches C, Bakker A, Gahr M, Ferguson-Smith MA, de Oliveira EH (2017). Comparative cytogenetics between two important songbirds: The zebra finch and the canary. PloS One.

[B11] dos Santos MS, Kretschmer R, Silva FA, Ledesma MA, O’Brien PCM, Ferguson-Smith MA, Garnero ADV, de Oliveira EH, Gunski RJ (2015). Intrachromosomal rearrangements in two representatives of the genus *Saltator* (Thraupidae, Passeriformes) and the occurrence of heteromorphic Z chromosomes. Genetica.

[B12] Ellegren H (2010). Evolutionary stasis: the stable chromosomes of birds. Trends Ecol Evol.

[B13] Ferguson-Smith MA, Trifonov V (2007). Mammalian karyotype evolution. Nat Rev Genet.

[B14] Furo IO, Monte AA, dos Santos MS, Tagliarini MM, O’Brien PCM, Ferguson-Smith MA, de Oliveira EH (2015). Cytotaxonomy of *Eurypyga helias* (Gruiformes, Eurypygidae): First karyotypic description and phylogenetic proximity with Rynochetidae. PLoS One.

[B15] Garnero AV, Gunski RJ (2000). Comparative analysis of the karyotype of *Nothura maculosa* and *Rynchotus rufescens* (Aves: Tinamidae) A case of chromosomal polymorphism. Nucleus.

[B16] Griffin DK, Robertson LBW, Tempest HG, Skinner BM (2007). The evolution of the avian genome as revealed by comparative molecular cytogenetic. Cytogenet Genome Res.

[B17] Guttenbach M, Nanda I, Feichtinger W, Masabanda JS, Griffin DK, Schmida M (2003). Comparative chromosome painting of chicken autosomal paints 1–9 in nine different bird species. Cytogenet Genome Res.

[B18] Hackett SJ, Kimball RT, Reddy S, Bowie RCK, Braun EL, Braun MJ, Chojnowski JL, Cox WA, Han KL, Harshman J (2008). A phylogenomic study of birds reveals their evolutionary history. Science.

[B19] Jarvis ED, Mirarab S, Aberer AJ, Li B, Houde P, Li C, Ho SY, Faircloth BC, Nabholz B, Howard JT (2014). Whole-genome analyses resolve early branches in the tree of life of modern birds. Science.

[B20] Kretschmer R, Gunski RJ, Garnero ADV, Furo IO, O’Brien PCM, Ferguson-Smith MA, de Oliveira EHC (2014). Molecular cytogenetic characterization of multiple intrachromosomal rearrangements in two representatives of the genus *Turdus* (Turdidae, Passeriformes). PLoS One.

[B21] Kretschmer R, de Oliveira EHC, dos Santos MS, Furo IO, O’Brien PCM, Ferguson-Smith MA, Garnero ADV, Gunski RJ (2015). Chromosome mapping of the large Elaenia (*Elaenia spectabilis*): evidence for a cytogenetic signature for passeriform birds?. Biol J Linn Soc.

[B22] Kretschmer R, Ferguson-Smith MA, de Oliveira EHC (2018). Karyotype evolution in birds: From conventional staining to chromosome painting. Genes.

[B23] Kretschmer R, de Oliveira Furo I, Gunski RJ, Garnero ADV, Pereira JC, O’Brien PCM, Ferguson-Smith MA, de Oliveira EHC, de Freitas TRO (2018). Comparative chromosome painting in Columbidae (Columbiformes) reinforces divergence in Passerea and Columbea. Chromosome Res.

[B24] Moyle RG, Slikas B, Whittingham LA, Winkler DW, Sheldon FH (2008). DNA sequence assessment of phylogenetic relationships among New World martins (Hirundinidae: *Progne*). Wilson J Ornithol.

[B25] Nanda I, Benisch P, Fetting D, Haaf T, Schmid M (2011). Synteny conservation of chicken macrochromosomes 1–10 in different Avian lineages revealed by cross-species chromosome painting. Cytogenet Genome Res.

[B26] Santos LP, Gunski RJ (2006). Revisão de dados citogenéticos sobre a avifauna brasileira. Rev Bras Ornitol.

[B27] Sasaki M, Ikeuchi T, Makino S (1968). A feather pulp culture technique for avian chromosomes, with notes on the chromosomes of the peafowl and the ostrich. Experientia.

[B28] Sheldon FH, Whittingham LA, Moyle RG, Slikas B, Winkler DW (2005). Phylogeny of swallows (Aves: Hirundinidae) estimated from nuclear and mitochondrial DNA sequences. Mol Phylogenet Evol.

[B29] Shibusawa M, Nishibori M, Nishida-Umehara C, Tsudzuk M, Masaband J, Griffin DK, Matsuda Y (2004). Karyotypic evolution in the Galliformes: An examination of the process of karyotypic evolution by comparison of the molecular cytogenetic findings with the molecular phylogeny. Cytogenet Genome Res.

[B30] Sigrist T (2013). Guia de campo Avis Brasilis: Avifauna Brasileira.

[B31] Tegelström H, Ryttman H (1981). Chromosomes in birds (Aves): evolutionary implications of macro- and microchromosome numbers and lengths. Hereditas.

[B32] Zhang G, Li C, Li Q, Li B, Larkin DM, Lee C, Storz JF, Antunes A, Greenwold MJ, Meredith RW (2014). Comparative genomics reveals insights into avian genome evolution and adaptation. Science.

